# Epilepsy surgery program in a resource‐limited setting in Vietnam: A multicentered collaborative model

**DOI:** 10.1002/epi4.12650

**Published:** 2022-10-05

**Authors:** Viet‐Thang Le, Minh‐An Thuy Le, Duc Hue Nguyen, Loc Ngoc Phuong Tang, Tuan Anh Pham, Anh Minh Nguyen, Minh Kien Nguyen, Tan Van Ngo, Thanh Trung Tran, Tuan Van Le, Pierre Jallon, Kheng‐Seang Lim

**Affiliations:** ^1^ Department of Neurosurgery University Medical Center at Ho Chi Minh City Ho Chi Minh City Vietnam; ^2^ Department of Neurosurgery University of Medicine and Pharmacy at Ho Chi Minh City Ho Chi Minh City Vietnam; ^3^ Department of Neurology University of Medicine and Pharmacy at Ho Chi Minh City Ho Chi Minh City Vietnam; ^4^ Department of Neurology Nguyen Tri Phuong Hospital Ho Chi Minh City Vietnam; ^5^ Department of Neurosurgery Nguyen Tri Phuong Hospital Ho Chi Minh City Vietnam; ^6^ Department of Neurology Pham Ngoc Thach University of Medicine Ho Chi Minh City Vietnam; ^7^ Department of Infection Disease Children Hospital 1 Ho Chi Minh City Vietnam; ^8^ Division of PET‐CT and Cyclotron, Nuclear Medicine Department Cho Ray hospital Ho Chi Minh City Vietnam; ^9^ Division of Neurology, Faculty of Medicine University of Malaya Kuala Lumpur Malaysia

**Keywords:** drug‐resistant, epilepsy surgery, limited‐resource, multidisciplinary team

## Abstract

**Objective:**

Although epilepsy surgery is more effective than medical therapy for drug‐resistant patients, it is underutilized in both high‐income and low‐ and middle‐income countries. In light of our efforts to establish an epilepsy surgery program in a resource‐limited setting, this study aimed to determine the outcome of the epilepsy surgery program in Ho Chi Minh City (HCMC), Vietnam.

**Methods:**

In 2018, we developed the HCMC epilepsy core multidisciplinary team with members from various hospitals and centers. The team typically included neurologists, neurosurgeons, neuropsychologists, psychiatrists, and nursing specialists. Presurgical evaluations were performed for patients with drug‐resistant epilepsy, fulfilling the ILAE criteria, with an epileptogenic lesion (mesial temporal sclerosis, low‐grade gliomas, or focal cortical dysplasia). All epilepsy surgeries were performed in two epilepsy surgery centers in HCMC between 2018 and 2021. The patients were followed up for at least 12 months.

**Results:**

Fifty‐two patients with drug‐resistant epilepsy underwent presurgical evaluation, of which 35 underwent surgery. Among the 52 patients, 20 (38.5%) underwent surgery after showing concordance among the results of standard presurgical assessments such as semiology, scalp interictal or ictal electroencephalography, and brain imaging. Among the 26 people with epilepsy who required more advanced evaluations, 15 underwent surgery with intraoperative electrocorticography to delineate the optimal resection borders. The outcomes of Engel Class I and Class II were achieved in 29/35 (82.8%) and 6/35 (17.2%) patients, respectively.

**Significance:**

The epilepsy surgery program with a multicentered collaborative model in a resource‐limited setting showed favorable outcomes in HCMC, Vietnam.

**Key Points**

- Although surgery is effective for epilepsy, it is underutilized in Vietnam.
- 35 of 52 drug‐resistant epilepsy patients (67.3%) undergoing presurgical evaluation proceeded with the surgery.
- 29/35 (82.8%) epilepsy surgery patients achieved the Engel I outcome postoperatively.
- A multicentered multidisciplinary approach is effective and practical in Vietnam with limited resources.

## 1 INTRODUCTION

### 1.1 Underutilization of epilepsy surgery

Epilepsy is one of the most common diseases of the nervous system that affects individuals of all ages and sexes worldwide. One meta‐analysis reported that the pooled incidence of epilepsy was 61.4/100 000 person‐years (95% confidence interval [CI] 50.7‐74.4). The incidence was three times higher in low‐ and middle‐income countries (139.0/100 000, 95% CI 69.4‐278.2) than in high‐income countries (48.9/100 000, 95% CI 39.0‐61.1).^1^ Although anti‐seizure medications (ASMs) are the most popular therapeutic agents for epilepsy, more than 30% of people with epilepsy (PWE) show incomplete seizure control with optimal medication.^2^ Epilepsy surgery has been reported to be more effective than medical therapy for drug‐resistant PWE.^3^ However, epilepsy surgery has been underutilized in high‐income countries,^4^, ^5^ and its utilization is even worse in low‐ and middle‐income countries.^6^ In Southeast Asian countries, although epilepsy surgery has been performed since the nineties, the availability of epilepsy centers, personnel, and presurgical evaluation tools, significantly more advanced diagnostic modalities, including single‐photon emission computed tomography (SPECT) and positron emission tomography (PET), is limited. In addition to these limiting factors, the lack of facilities, expertise, and funding have been the essential obstacles to the adequate adoption of epilepsy surgery.^6^

Successful surgery requires a multidisciplinary team, including trained epileptologists, neurosurgeons, electroencephalography (EEG) technicians, and other specialties, and presurgical evaluation is an essential step to ensure successful surgical outcomes. Ideal candidates for surgery can be identified by a combination of seizure semiology and essential diagnostic tools, including brain magnetic resonance imaging (MRI) and long‐term monitoring video EEG.^7^ In cases of lesion‐induced epilepsy, surgery can be performed using semiology, video scalp EEG, and brain MRI with an epilepsy protocol. According to the Committee to Revise the Guidelines for Services, Personnel, and Facilities at Specialized Epilepsy Centers, a specialized epilepsy center is required to provide routine care and comprehensive diagnostic and management services to patients with uncontrolled seizures (such as drug‐resistant epilepsy).^8^

### 1.2 Epilepsy surgery program in Vietnam

Vietnam is a low–middle‐income country in the ASEAN organization with a population of 97 million people. In Vietnam, the age‐adjusted incidence of epilepsy was 44.8 per 100 000 (95% CI 30.6‐59.0).^9^ Epilepsy has long been considered to be a psychiatric disorder. The diagnosis of epilepsy is often accompanied by lifelong ASM therapy. In the past, resective surgery for brain tumors was offered in some cases and on an individual approach by neurosurgeons in a few hospitals in Vietnam. Still, epilepsy surgery and multidisciplinary collaboration were unavailable.

In 1997, approximately 60 neurosurgeons were working in Vietnam. However, neurosurgical training has expanded dramatically in the past 20 years. In 2017, the Asian and Oceanian Association of Neurology (AOAN) survey identified 800 neurologists and 300 neurosurgeons, and the estimated number of neurologists per 100 000 population was 0.86.^10^

#### 1.2.1 Expertise

In Vietnam, residency programs for neurology and neurosurgery are available after completing the basic medical degree; however, training in subspecialties, including epilepsy, is not available.^10^ To develop an epilepsy surgery program, neurologists and neurosurgeons were trained as epilepsy fellows in foreign experienced epilepsy centers in Malaysia, Taiwan, and Korea. The tuition fee for training was obtained from various international scholarships, such as the Asian Epilepsy Academy (ASEPA) scholarship or the Korean Epilepsy Society or was funded by local hospitals (University Medical Center [UMC], Nguyen Tri Phuong hospital [NTP]). The UMC epilepsy unit was established in 2015, and an epilepsy surgery team was built in 2018. The Epilepsy Monitoring Unit (EMU) in NTP was established in 2018. One survey in 2018 identified nine epilepsy centers in Vietnam; however, no level 4 center can perform adequate presurgical evaluation and invasive procedures.^6^ Care of patients at specialized epilepsy centers is provided by a collaborative multidisciplinary team directed by an epileptologist or a neurosurgeon with expertise in epilepsy.^8^

**FIGURE 1:**
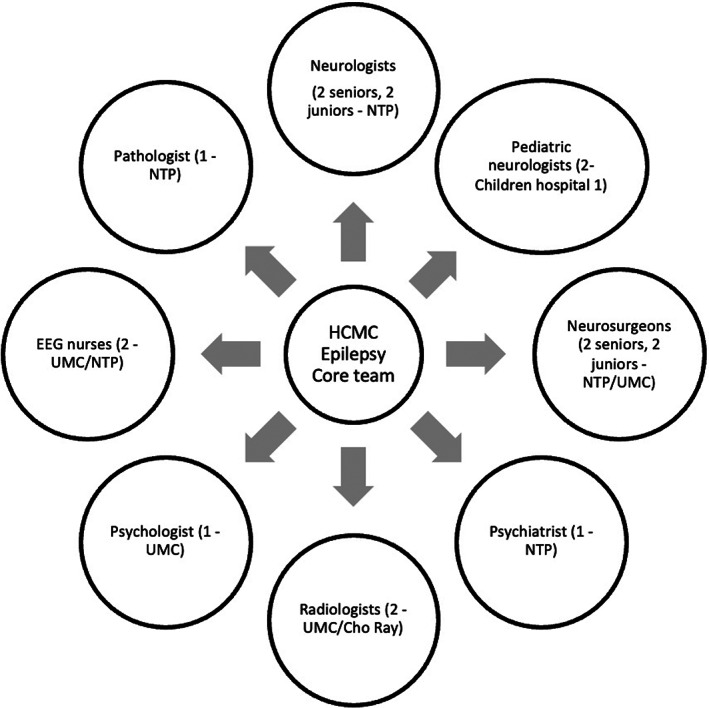
HCMC core team personnel (the number of physicians involved – Hospital)

Although several studies were published about epilepsy surgery in limited‐resource countries, the authors reported the development of every single epilepsy center.^11^, ^12^, ^13^, ^14^ In HCMC, developing a multidisciplinary team in each center or hospital was impossible because of the lack of expertise and personnel, which was the most significant barrier to this objective in Vietnam.^6^ Therefore, in 2018, we developed the HCMC epilepsy core team, a multidisciplinary team from various hospitals and centers in HCMC, including NTP, UMC, Children's Hospital, and Cho Ray Hospital (Figure 1). This model can overcome the existing barriers by uniting specialists from different hospitals, including neurologists, neurosurgeons, pediatric neurologists, psychiatrists, radiologists, and other experts. Internet‐based teleconferences between the epilepsy team and international epileptologists were organized to discuss complicated surgery candidates.

#### 1.2.2 Facilities

Specialized equipment and facilities for presurgical evaluation are needed to develop an epilepsy surgery program. Among the standard modalities, both long‐term video EEG and brain MRI were available on‐site in each hospital (NTP and UMC). An advanced modality, PET scan, is only available at Cho Ray Hospital, HCMC. Intraoperative electrocorticography (ECoG) was introduced in 2020. In light of our efforts to establish an epilepsy surgery program in a resource‐limited setting, this study aimed to determine the outcome of the epilepsy surgery program with a multicentered core team model in HCMC.

## 2 METHODS

### 2.1 Study design

This was a retrospective observational study conducted between 2018 and 2021.

### 2.2 Participants

Candidates for presurgical evaluation were PWE with drug resistance that fulfilled the ILAE criteria;^15^ spatial relation of the lesion to the epileptogenic zone varies according to the nature of the epileptogenic lesion (mesial temporal sclerosis (MTS), low‐grade gliomas (LGGs), or focal cortical dysplasia (FCD)). We excluded patients with negative MRI findings and epilepsy with stroke or traumatic origin.

### 2.3 Procedures

#### 2.3.1 Presurgical evaluation protocol

Since Vietnam is a resource‐limited country, we adapted the presurgical evaluation protocol used in UMMC, Malaysia, with permission.^12^ Trained neurologists evaluated PWE diagnosed with drug‐resistant epilepsy (DRE) based on clinical information, including age at onset, semiology, number of ASMs, medical history, and primary modalities such as video EEG and brain MRI. MRI was performed with an epilepsy protocol,^16^ while long‐term video EEG with a duration of 1‐3 days was conducted to capture interictal discharges and/or clinical seizures.^17^ At the beginning of the program, before assessing the probability of successful surgery, we chose cases showing lesional MRI with surgically remediable epilepsy syndromes, including MTS, LGGs, and FCD, to discuss in the team conference. The team conferences were organized monthly or every two months. The core team reviewed clinical, EEG, and neuroimaging data at the meeting. The decision was made to include either a surgical plan or add indications for more advanced examinations, including FDG‐PET scans, fMRI, and diffusion tensor imaging (DTI). Morphometric analysis program (MAP)^18^ was completed with assistance from Malaysia. After advanced evaluations, another conference was held to discuss the surgical decisions.

#### 2.3.2 Surgical planning

In cases showing concordant findings for at least three examinations, including semiology, interictal EEG, ictal EEG, and brain MRI, and lesions that did not affect the eloquent cortex, the surgical plan was anterior temporal lobectomy (ATL) for MTS or lesionectomy for FCD or LGGs. The decision to perform surgery was also made if the potential epileptogenic zone was confirmed and did not overlap the eloquent cortex after additional advanced examinations such as FDG‐PET, fMRI, or MAP. In some cases with cortical lesions, intraoperative ECoG was performed to optimize the surgical borders.

### 2.4 Variables

The demographic characteristics collected included sex, age at onset, and age at surgery. In addition, the clinical features included MRI abnormalities (single or multiple), epileptogenic zone (temporal or extratemporal), type of surgery (anterior temporal lobectomy or lesionectomy), and neuropathology (LGGs,^19^ hippocampal sclerosis [HS], or FCD).

Postoperative follow‐up assessments of the PWE were conducted for at least one year at the epilepsy clinic in either UMC or NTP with the same medication regimen as before surgery. The postoperative evaluation was clinical examinations, seizure frequency diaries, and post‐operative MRI and EEG after 2 years to consider ASM reduction. The surgical outcome was categorized into four classes using the Engel outcome scale.^20^ Class I patients were free of disabling seizures. Patients with only simple, focal, non‐disabling seizures and/or without seizures in an 8‐month period or more at the time of the latest assessment were included in this category. Class II was defined as one who seldom experienced seizures at a frequency of three or less per year. Class III corresponded to a “worthwhile” result characterized by a reduction in seizure frequency or intensity that improves the patient's quality of life. In contrast, patients whose seizure frequency reduction was insignificant and did not improve daily functioning were assigned to class IV.

### 2.5 Statistical methods

Data entry and statistical analyses were conducted using Epidata and SPSS version 22.0, respectively. Descriptive statistics were used to evaluate the distribution of scores (mean, range, standard deviation [SD], and standard error [SE]).

## 3 RESULTS

### 3.1 Presurgical evaluation

**FIGURE 2:**
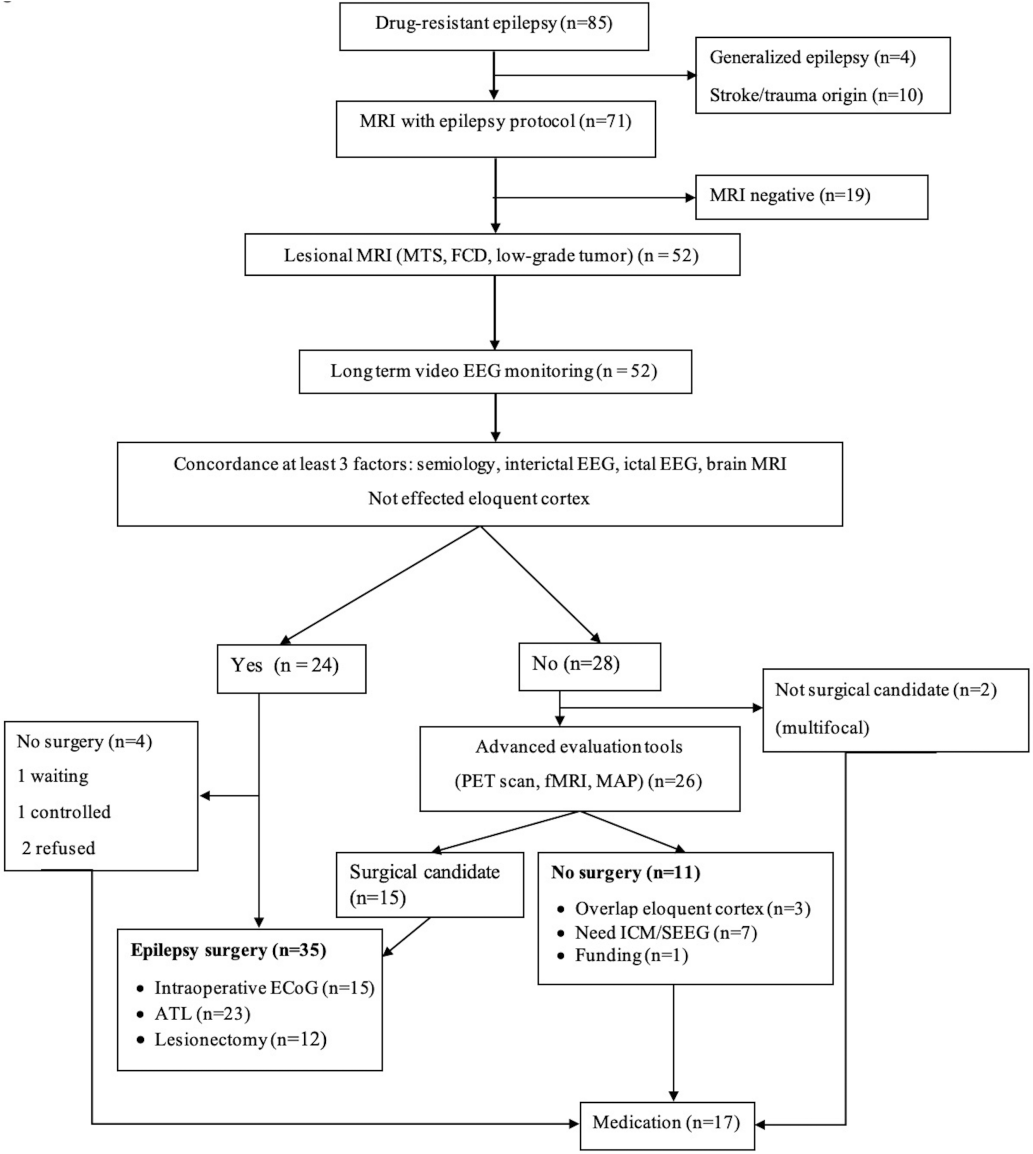
Presurgical evaluation flow chart. ATL, anterior temporal lobectomy; ECoG, electrocorticography; EEG, electroencephalography; FCD, focal cortical dysplasia; fMRI, functional magnetic resonance imaging; ICM, intracranial monitoring; MAP, morphometric analysis program; MRI, magnetic resonance imaging; MTS, mesial temporal sclerosis; PET, positron emission tomography; SEEG, stereoelectroencephalography

From 2018 to 2021, 85 DRE patients were evaluated in this study. After considering exclusion criteria, 52 patients with DRE underwent presurgical evaluation. The details are shown in Figure 2. Based on concordance among standard evaluations, including semiology, scalp video EEG, and brain imaging, 20/52 (38.5%) patients underwent surgery. Among the 26 PWE who required more advanced evaluations, we performed PET scan in one case with left temporal lobe epilepsy syndrome, concordant interictal EEG, and a delicate lesion of the left temporal cortex showed FCD. Interictal FDG‐PET scan shows hypometabolism in the left temporal lobe. Intraoperative ECoG recordings were first introduced at our centers in 2020 and were conducted on 15 patients. In our study, ECoG was helpful in tailoring the margins of the epileptogenic zone, especially for those undergoing extratemporal lobe epilepsy resections (7 patients).

### 3.2 Epilepsy surgery outcomes

Among the surgical candidates, 21 were female, and 14 were male, with a mean age of 38.1. In terms of surgery type, 23 (65.7%) patients underwent anterior temporal lobectomy (ATL), and 12 (34.3%) experienced lesionectomy. The most common etiological factor for drug‐resistant epilepsy was LGGs: low‐grade astrocytoma (15), ganglioglioma (2), and dysembryoplastic neuroepithelial tumors (1), which were observed in 18 patients (51.4%). The other common etiologies were MTS in 14 patients (40.0%) and cortical malformations in two patients (5.8%) (Table 1).

**TABLE 1:** Demographic and clinical characteristics of epilepsy surgery cases

Characteristic	N	%
Female	21	60.0
Left‐sided surgery	18	51.4
Mean age of onset, years ± SD (range)	25.2 ± 11.7 (3‐46)	
Mean age of surgery, years ± SD (range)	38.1 ± 10.1 (21‐53)	
Mean seizure frequency/month ± SD (range)	15.5 ± 62.3 (6‐240)	
MRI findings		
Single abnormalities	34	97.2
Multiple abnormalities	1	2.8
Epileptogenic zone		
Temporal lobe	28	80.0
Extratemporal lobe	7	20.0
Surgery		
ATL	23	65.7
Lesionectomy	12	34.3
Extratemporal	7	20.0
Temporal	5	14.3
ECoG		
Temporal	8	22.8
Extratemporal	7	20.0
Neuropathology		
Low‐grade astrocytoma	15	42.8
Hippocampal sclerosis	14	40.0
Ganglioglioma	2	5.8
Focal cortical dysplasia	2	5.8
DNET	1	2.8
Chronic encephalitis	1	2.8
The mean numbers of ASMs ± SD		
At the time of surgery	2.85 ± 0.9	
At last follow‐up (at least 1 year)	0.91 ± 0.8	

Abbreviation: SD, Standard deviation.

In the assessment of postoperative outcomes, 29 patients showed class I Engel outcomes after a mean follow‐up period of 18 months (range, 12‐31 months). Six patients had rare seizures (class II), and none showed class III or IV outcomes. Among intraoperative ECoG cases, all cases achieved favorable outcome, including Engel Class I (80.0%) and Engel Class II (20.0%). No other morbidities or mortalities occurred in the 35 patients (Table 2).

**TABLE 2:** Post‐operative outcomes based on the multimodality assessment

Characteristic	Engel outcome scale			
	Class I	Class II	Class III	Class IV
Temporal lobe epilepsy				
Semiology + interictal‐ictal EEG + MRI	15	3	0	0
Semiology + interictal EEG + MRI	7	2	0	0
Semiology + interictal‐ictal EEG + MRI + PET scan	1	0	0	0
Extratemporal lobe epilepsy				
Semiology + interictal‐ictal EEG+ MRI	6	1	0	0
Total	29	6	0	0
EcoG (n = 15)				
Temporal	6	2	0	0
Extratemporal	6	1	0	0
Total	12	3	0	0

## 4 DISCUSSION

### 4.1 Epilepsy surgery outcomes

Engel Class I and Class II outcomes over at least 1 year of follow‐up were achieved in 82.8% and 17.2% of the patients in our study, respectively. This result was similar to previous studies reported in children and adults, although the seizure‐free percentage was higher after temporal lobectomy with MTS (60%‐90%).^3^, ^21^ We found that comparable neuropathologies (glioma, DNET, cavernous hemangioma, HS, or FCD) in the medial temporal and temporal cortex of patients undergoing ATL were associated with similar seizure‐free rates.^22^ Shih et al. reported no difference in postoperative outcome between those who underwent anterior temporal lobe resection, temporal lobe resection, or a combination of cortical resection and anterior temporal lobe resection in temporal lobe epilepsy. No postoperative mortality was recorded due to modern surgical techniques and facilities, effective anesthesia and resuscitation, active postoperative monitoring at the neurosurgery unit, and postoperative CT scans for early detection of postoperative hematoma. These results indicate that the strategy has been effective despite limited resources, especially in the context of lesion‐positive MRI. In our newly established surgery program, we intentionally chose typical and straightforward candidates to gain experience for more complicated cases in the future, such as lesion‐absent or multifocal epilepsy.

### 4.2 Strategies for resource‐limited countries

This study attempted to address some issues encountered while developing our epilepsy surgery program in Vietnam, a resource‐limited country. The decision‐making step requires a multidisciplinary approach in which different investigators collaborate to understand epileptology. This also involves knowledge about when activities should not be performed and require additional assessment, which is essential in identifying beneficiaries with available resources. We have already discussed several challenges in providing optimal surgical management for people with medically refractory epilepsy in such regions.^6^ Here, we also attempt to answer the challenging questions during the establishment of our epilepsy surgery program.

First, a strength of this study is the interdisciplinary approach to the management of individuals with drug‐resistant epilepsy. The primary investigations were to perform the MRI modality with epilepsy protocol and to record the long‐term video EEG, which was investigated in DRE patients after standard investigations to determine the location and lateralization process. Advanced tools such as interictal FDG‐PET and intraoperative ECoG can be used case‐by‐case after a team conference or teleconference with foreign experts. Based on those findings and patient suitability, they are finally taken up for surgery or undergo other procedures. The utilization of human and material resources from various centers and hospitals solved the challenges related to a lack of expertise and facilities. As mentioned above, we developed a multicenter core team that has been a unique concept in Vietnam since 2018. Establishing a collaborative partnership among different centers, including international collaboration, may give access to rare expertise and expensive diagnostic tools.

Expertise is the most essential factor when developing a surgery program. Training epileptologists and neurosurgeons who can create and manage a team could be critical to developing a successful epilepsy surgery program. Trained epileptologists help to reduce the risk of missing crucial clinical information, such as seizure type, associated comorbidities, and the influence of uncontrolled seizures, and trained neurosurgeons reduce surgery‐related comorbidity and mortality. Furthermore, radiologists with experience in epilepsy cases help to find subtle abnormalities such as FCD. In Vietnam, training in subspecialties such as epilepsy is not provided. We sought help from well‐established and specialized epilepsy centers in other developed countries to achieve this goal. Other specialists such as neuropathologists and psychologists have also been trained for the team to optimize advanced epilepsy care. International collaboration via teleconference and visiting professorships (Taiwan, Malaysia, and France) was another solution for overcoming expertise‐related barriers.

The next step in developing a multicentered effective surgery program is utilizing primary presurgical modalities and advanced, expensive tools from other centers. A realistic preoperative protocol is essential to choosing appropriate surgical candidates. In HCMC, every single center lacks funding to equip all evaluation technologies; sharing available resources from different centers is a reasonable model. A practical protocol focused on standardizing the primary modalities, including video EEG and MRI with epilepsy protocol. We also utilized FDG‐PET scan, which was not previously used in epilepsy assessment. A multicentered epilepsy team is affordable for patients and health systems.

### 4.3 Limitations

This study had some limitations. The small sample size compared with a large number of potential surgical candidates in the population highlighted the necessity of a standard referral system in the future. At the early stage, our team selected straightforward cases to gain experience. We excluded patients with negative MRI findings, those with stroke or traumatic origin, and multiple epileptogenic zones in both hemispheres. The number of patients under presurgical evaluation will be improved in the future with more complex cases. Another limitation was the short follow‐up period after surgery. The follow‐up period should be extended in future studies to clarify the long‐term prognosis.

## 5 CONCLUSION

An epilepsy surgery program with a multicentered collaborative model showed favorable outcomes in HCMC, Vietnam. Sharing expertise and modalities among epilepsy centers is essential. This study provided initial evidence for the effectiveness of epilepsy surgery with a realistic presurgical protocol in a resource‐limited country.

## AUTHOR CONTRIBUTIONS

VTL and MATL designed this study. MATL and LNPT performed literature searches. VTL and DHN collected data, and MATL and VTL wrote the initial draft of the manuscript. All listed authors revised the final manuscript.

## CONFLICT OF INTEREST

None of the authors has any conflict of interest to disclose.

## ETHICAL STATEMENT

We confirm that we have read the Journal's position on issues involved in ethical publication and affirm that this report is consistent with those guidelines.

## References

[1] Fiest KM , Sauro KM , Wiebe S , Patten SB , Kwon CS , Dykeman J , et al. Prevalence and incidence of epilepsy: a systematic review and meta‐analysis of international studies. Neurology. 2017;17(88):296–303. 10.1212/WNL.0000000000003509 PMC5272794 27986877

[2] Kwan P , Brodie MJ . Early identification of refractory epilepsy. N Engl J Med. 2000;342:314–9. 10660394 10.1056/NEJM200002033420503

[3] Wiebe S , Blume WT , Girvin JP , Eliasziw M . A randomized, controlled trial of surgery for temporal‐lobe epilepsy. N Engl J Med. 2001;345:311–8. 11484687 10.1056/NEJM200108023450501

[4] Burneo JG , Jette N , Theodore W , Begley C , Parko K , Thurman DJ , et al. Disparities in epilepsy: report of a systematic review by the north American Commission of the International League against Epilepsy. Epilepsia. 2009;50:2285–95. 19732134 10.1111/j.1528-1167.2009.02282.x PMC3181115

[5] Englot DJ , Ouyang D , Garcia PA , Barbaro NM , Chang EF . Epilepsy surgery trends in the United States, 1990‐2008. Neurology. 2012;17(78):1200–6. 10.1212/WNL.0b013e318250d7ea PMC3324320 22442428

[6] Le M‐AT , Fong S‐L , Lim K‐S , Gunadharma S , Sejahtera DP , Visudtibhan A , et al. Underutilization of epilepsy surgery in ASEAN countries. Seizure. 2019;69:51–6. 30974407 10.1016/j.seizure.2019.04.002

[7] Asadi‐Pooya AA , Sperling MR . Strategies for surgical treatment of epilepsies in developing countries. Epilepsia. 2008;49:381–5. 17941843 10.1111/j.1528-1167.2007.01383.x

[8] Labiner DM , Bagic AI , Herman ST , Fountain NB , Walczak TS , Gumnit RJ , et al. Essential services, personnel, and facilities in specialized epilepsy centers–revised 2010 guidelines. Epilepsia. 2010;51:2322–33. 20561026 10.1111/j.1528-1167.2010.02648.x

[9] Tuan NA , Cuong le Q , Allebeck P , Chuc NT , Persson HE , Tomson T . The incidence of epilepsy in a rural district of Vietnam: a community‐based epidemiologic study. Epilepsia. 2010;51:2377–83. 20726874 10.1111/j.1528-1167.2010.02699.x

[10] Roxas A Jr , Mehndiratta MM , Bornstein N , Macdonell R , Lim KS , Ng PW , et al. The professional practice and training of neurology in the Asian and Oceanian region: a cross‐sectional survey by the Asian and Oceanian Association of Neurology (AOAN). J Neurol Sci. 2017;15(382):108–15. 10.1016/j.jns.2017.09.022 29111001

[11] Muttaqin Z. Epilepsy surgery in Indonesia: achieving a better result with limited resources. 2013.

[12] Lim KS , Ahmad SB , Narayanan V , Kartini R , Ramli NM , Mun KS , et al. editorsLevel 4 comprehensive epilepsy program in Malaysia, a resource‐limited country. Neurology Asia. 2017;22(4):299–305.

[13] Li W , Hao N , Liu W , An D , Yan B , Li J , et al. The experience of the multidisciplinary team in epilepsy management from a resource‐limited country. Epilepsia Open. 2018;4:85–91. 30868118 10.1002/epi4.12290 PMC6398094

[14] Radhakrishnan K . Challenges in the management of epilepsy in resource‐poor countries. Nat Rev Neurol. 2009;5:323–30. 19455183 10.1038/nrneurol.2009.53

[15] Kwan P , Arzimanoglou A , Berg AT , Brodie MJ , Allen Hauser W , Mathern G , et al. Definition of drug resistant epilepsy: consensus proposal by the ad hoc task force of the ILAE commission on therapeutic strategies. Epilepsia. 2010;51:1069–77. 19889013 10.1111/j.1528-1167.2009.02397.x

[16] Ramli N , Rahmat K , Lim KS , Tan CT . Neuroimaging in refractory epilepsy. Current practice and evolving trends. Eur J Radiol. 2015;84:1791–800. 26187861 10.1016/j.ejrad.2015.03.024

[17] Lim KS , Fong SL , Thuy Le MA , Ahmad Bazir S , Narayanan V , Ismail N , et al. 48‐hour video‐EEG monitoring for epilepsy presurgical evaluation is cost‐effective and safe in resource‐limited setting. Epilepsy Res. 2020;162:106298. 32172144 10.1016/j.eplepsyres.2020.106298

[18] Huppertz HJ , Kurthen M , Kassubek J . Voxel‐based 3D MRI analysis for the detection of epileptogenic lesions at single subject level. Epilepsia. 2009;50:155–6. 19125836 10.1111/j.1528-1167.2008.01734.x

[19] Tanriverdi T , Kemerdere R , Baran O , Sayyahmelli S , Ozlen F , Isler C , et al. Long‐term surgical and seizure outcomes of frontal low‐grade gliomas. Int J Surg. 2016;33:60–4. 27475744 10.1016/j.ijsu.2016.07.065

[20] Engel J Jr . Surgery for seizures. N Engl J Med. 1996;334:647–52. 8592530 10.1056/NEJM199603073341008

[21] Englot DJ , Wang DD , Rolston JD , Shih TT , Chang EF . Rates and predictors of long‐term seizure freedom after frontal lobe epilepsy surgery: a systematic review and meta‐analysis. J Neurosurg. 2012;116:1042–8. 22304450 10.3171/2012.1.JNS111620

[22] Shih YH , Lirng JF , Yen DJ , Ho DM , Yiu CH . Surgery of intractable temporal lobe epilepsy presented with structural lesions. J Chin Med Assoc. 2003;66:565–71. 14703272

